# Identity-by-Descent Mapping to Detect Rare Variants Conferring Susceptibility to Multiple Sclerosis

**DOI:** 10.1371/journal.pone.0056379

**Published:** 2013-03-05

**Authors:** Rui Lin, Jac Charlesworth, Jim Stankovich, Victoria M. Perreau, Matthew A. Brown, ANZgene Consortium, Bruce V. Taylor

**Affiliations:** 1 Menzies Research Institute Tasmania, University of Tasmania, Hobart, Australia; 2 Walter and Eliza Hall Institute of Medical Research, Melbourne, Australia; 3 Centre for Neurosciences, Department of Anatomy and Neuroscience, University of Melbourne, Melbourne, Australia; 4 University of Queensland Diamantina Institute, Brisbane, Australia; Ohio State University Medical Center, United States of America

## Abstract

Genome-wide association studies (GWAS) have identified around 60 common variants associated with multiple sclerosis (MS), but these loci only explain a fraction of the heritability of MS. Some missing heritability may be caused by rare variants that have been suggested to play an important role in the aetiology of complex diseases such as MS. However current genetic and statistical methods for detecting rare variants are expensive and time consuming. ‘Population-based linkage analysis’ (PBLA) or so called identity-by-descent (IBD) mapping is a novel way to detect rare variants in extant GWAS datasets. We employed BEAGLE fastIBD to search for rare MS variants utilising IBD mapping in a large GWAS dataset of 3,543 cases and 5,898 controls. We identified a genome-wide significant linkage signal on chromosome 19 (LOD = 4.65; p = 1.9×10^−6^). Network analysis of cases and controls sharing haplotypes on chromosome 19 further strengthened the association as there are more large networks of cases sharing haplotypes than controls. This linkage region includes a cluster of zinc finger genes of unknown function. Analysis of genome wide transcriptome data suggests that genes in this zinc finger cluster may be involved in very early developmental regulation of the CNS. Our study also indicates that BEAGLE fastIBD allowed identification of rare variants in large unrelated population with moderate computational intensity. Even with the development of whole-genome sequencing, IBD mapping still may be a promising way to narrow down the region of interest for sequencing priority.

## Introduction

Multiple sclerosis (MS) is a complex neurological disease of the central nervous system (CNS) triggered by environmental and genetic factors. There is considerable evidence for a significant genetic component to MS susceptibility, such as a higher concordance rate in monozygotic twins (24%–30%) than dizygotic twins (3%–5%) [1], [2]. As for other immune diseases, genome-wide association studies (GWAS) have been highly successful for MS: uncovering around 60 common genetic variants associated with disease [3]–[13]. The majority of these variants lie near genes with known functions in the immune system and these variants have also been associated with other autoimmune diseases, often in the opposite direction [14]. Virtually all of the variants confer modest increases in disease risk, the outstanding exception being the strong association with the HLA-DRB1*15:01 allele in the major histocompatibility complex (MHC), which was first detected in the 1970's [15], [16].

Despite this success, the variants identified by GWAS to date only explain 18–24% of the heritability of MS [13], [17]. While much of the missing heritability is probably explained by common variants of even smaller effect sizes, some heritability may be explained by rare variants of larger effect size. Standard analysis of GWAS data is not designed to detect associations with rare variants that many believe may play an important role in the aetiology of complex traits [18]–[20]. Interestingly, GWAS have had less success for putative neurodegenerative diseases, such as Parkinson's disease, than for MS. For these diseases, family-based approaches detecting rare variants have been more successful [21], [22]. This raises the possibility that rare variants under negative selection pressure are relatively more important in the genetic architecture of neurodegenerative processes, whereas common variants under balancing selection are more important in the genetic architecture of immunological processes. Discovery of rare MS susceptibility variants may alter perspectives on the relative importance of immunological & neurodegenerative processes in MS onset.

Standard analyses of GWAS data are not designed to detect associations with low frequency variants (MAF≤5%), and other strategies are required. One approach is to re-sequence loci containing common susceptibility variants identified from GWAS studies. This strategy was used to detect rare variants in *IFIH1* conferring protection to type I diabetes [18]. However this strategy precludes the identification of new loci. Eventually it will be possible to overcome this limitation by whole genome sequencing, but it remains prohibitively expensive to perform adequately-powered studies. An alternative is to re-analyse GWAS data using identity-by-descent (IBD) mapping [23], also referred to as ‘population-based linkage analysis’ (PBLA) [24]. PBLA describes linkage analysis applied at the population level to detect mega base-scale regions where cases have inherited long haplotypes from distant ancestors, 10–100 generations ago. IBD mapping is performed on the unrelated individuals to determine whether these mega-base scale regions are identical and inherited from a common ancestor. If the common ancestor lived more than ten generations ago the individuals will share very short tracts of genetic material, and a shared haplotype that is very rare is also very likely to be IBD. HapMap Phase 3 identified that lower frequency variants should, on average, be younger than more common variants; and thus display a greater extent of haplotype sharing [25]. Therefore, if case pairs can be detected with long shared haplotypes (generally one to five megabases) inherited from distant common ancestors, then rare variants influencing disease risk can be localised. Even when whole genome sequencing becomes cheap enough to pursue with substantial sample sizes, IBD mapping may still help reduce the massive multiple testing problem by prioritizing regions. This is similar to the technique of prioritising association signals in regions of linkage [26].

Several methods of IBD mapping have been published: these include PLINK [24], GERMLINE [27], BEAGLE IBD [28] and BEAGLE fastIBD [29]. The models employed by PLINK and GERMLINE assume SNPs are in linkage equilibrium, and so ‘pruning’ of SNPs [24] is required to avoid false positives due to under-estimates of population haplotype frequencies. However pruning of SNPs in incomplete linkage disequilibrium (LD) discards potentially useful information and reduces power. BEAGLE IBD and fastIBD implement a variable length Hidden Markov Model [30] to account for LD and model haplotype frequencies more accurately. BEAGLE fastIBD runs considerably faster than BEAGLE IBD (of the order of 1000 times faster with large GWAS datasets). This is mainly because 1) it does not formally model IBD status (‘IBD’/’not IBD’) between pairs of individuals using a Hidden Markov Model as in BEAGLE IBD; 2) it stores haplotype frequencies in a data dictionary (as in GERMLINE) which means computational time scales with sample size *n* like *n* log *n* instead of *n^2^*.

To detect MS rare variants, we here use BEAGLE fastIBD to perform an IBD analysis on several large MS GWAS datasets comprised 3543 cases and 5898 controls. We identified a region of high significance on chromosome 19q13.43, with a genome-wide significant localisation signal (p = 1.9×10^−6^; LOD = 4.65) using thresholds based on IBD segment length greater than 3 cM and the probability p-value less than 10^−9^ (3cM_1e-9). This locus was deemed genome-wide significant according to the recently established genome-wide significance thresholds set for IBD mapping [31]. Analysis of expression data and investigation of genes in this area support the hypothesis for regulation of gene expression in this region to impact upon development or health of CNS tissue. Our analyses also illustrate some of the practical issues to deal with in IBD analyses, and demonstrate that IBD mapping can form a potentially powerful method for detecting rare variants in unrelated individuals at the population level.

## Methods

### Study subjects

All the MS cases and controls were recruited and genotyped from MS GWAS totaling 3,543 cases and 5,898 controls. Of these, 1,618 cases and 3,413 controls were from an Australian and New Zealand MS GWAS conducted by the Australian and New Zealand Multiple Sclerosis Genetics Consortium (ANZgene) [3], and those DNA samples were genotyped on the Illumina Infinium Hap370CNV array [3]. An additional 861 Australian and New Zealand MS cases were genotyped with the Illumina Human660-Quad chip as part of a GWAS performed by the International MS Genetics Consortium (IMSGC) and the Wellcome Trust Case Control Consortium-2 (WTCCC2) [13]. Controls included 1,531 unrelated Australian samples from a GWAS genotyped by Queensland Institute of Medical Research (QIMR) with the Illumina Human610-Quad chip [32], and 1064 MS cases and 954 controls genotyped with the Sentrix® HumanHap550 BeadChip from a GWAS conducted in the US (GeneMSA) [6] [accessed via dbGAP].

### Quality control of data

Conservative quality control measures were imposed both on the individual datasets before merging, and in the combined dataset after merging: SNPs with call rates less than 0.95 or in Hardy-Weinberg disequilibrium (p<10^−7^) were discarded, as were samples with call rates less than 0.98. Duplicates and close relatives were also removed. This data cleaning was performed using PLINK.

A principal components analysis (PCA) was conducted by EIGENSTRAT [33] to exclude ancestry outliers and examine population structure within the remaining samples. First, SNPs in strong LD were pruned (using the PLINK – indep command with options 50 5 1.5), and then we excluded previously identified regions of high LD [34]. Outliers in the PCA were excluded using standard settings in Eigenstrat (more than six standard deviations from the mean along the first 10 principal components). All chromosomal locations refer to Human genome version hg18.

### Running BEAGLE fastIBD and results processing

The fastIBD analyses were conducted using BEAGLE (http://faculty.washington.edu/browning/beagle/). In brief, genotypes for the merged, cleaned dataset were converted to BEAGLE format by using the linkage2Beagle.jar utility program. We then used the BEAGLE method for phasing the data and identifying IBD segments simultaneously, using the ‘fastibdthreshold’ option. This procedure was run 10 times for each chromosome starting with different seeds of the random number generator.

The output of these calculations was a series of “putative” IBD segments shared between pairs of individuals. Each segment comes with the following information attached: ids for the pair of individuals, first and last SNPs in the IBD segment, length of the segment in centimorgans, and probability of the two individuals both carrying the segment if it was not IBD. We filtered these segments using various maximum probabilities and minimum segment lengths, as recommended in the BEAGLE manual. Results from the 10 runs were combined by taking the union of IBD segments detected in each run. From the final list of segments, we wrote a Perl script to count numbers of case-case pairs (*yi*), case-control pairs (*ui*) and control-control pairs (*vi*) estimated to share haplotypes IBD at each SNP.

### Analysis of IBD

We focused on the detection of loci where groups of cases have inherited rare susceptibility alleles IBD. To do this, we modelled IBD sharing *yi* in case-case pairs (“case pairs”) as a function of IBD sharing in *xi*  =  *ui* + *vi* in case-control pairs and control-control pairs combined (“control pairs”).

We tried various methods to model the *yi* as a function of the *xi*: linear regression, negative binomial regression and Poisson regression. Models were fitted using R [35] and goodness of fit was assessed by examining diagnostic plots (**SR_commands S1**).

At SNPs *i* with more IBD sharing in cases than expected, residuals *zi* from the fitted models should be large and positive. To present residuals on a scale more familiar to geneticists, we converted them to LOD scores using the formula LOD*i*  =  *zi* ^2^/(2*log_e_(10).

At the SNPs with the highest LOD scores, we calculated the proportions of case pairs sharing IBD in various populations, and plotted networks of case and control pairs sharing IBD with each other using the R network package (http://cran.rproject.org/web/packages/network/index.html).

## Results

### Study samples from GWAS after cleaning

11 individuals were excluded due to call rates less than 0.98 and an additional 202 individuals were excluded because they were close relatives or duplicates. PCA was conducted on a subset of 77,856 SNPs not in LD, which were common to all sample sets. Through successive iterations 251 outliers (37 AUS cases; 9 AUS controls; 6 UK controls; 97 US cases; 102 US controls) were excluded. All datasets overlapped well after the removal of outliers (**Figure S1**). In summary, following cleaning there were 3,243 cases and 5,725 controls with 274,735 autosomal SNPs in the final analysis (**Table 1**).

**Table 1:** Sample numbers from GWAS (after cleaning).

GWAS dataset	Country of origin		No. Case	No. Control	Total	No. SNPs
	Cases	Controls				
ANZgene [3]	AUS, NZ	US, UK	1,608	3,404	5,012	300,900
WTCCC2 [13]	AUS	-	766	-	766	586,393
QIMR [32]	-	AUS	-	1,516	1,516	529,292
GeneMSA [6]	US	US	878	805	1,683	550,677
Total			3,252	5,725	8,977	274,735*

* The number of SNPs that passed QC in all 4 GWAS datasets.

AUS  =  Australia, NZ = New Zealand.

### Results of IBD analysis

We detected IBD with the threshold of IBD segment greater than 3 cM and the haplotype probability p-value less than 10^−9^ (3cM_1e-9). A strong linkage signal was observed in the HLA region (LOD = 3.58), while the strongest signal in non-HLA region was on chromosome 19 (LOD = 4.65), which reached genome-wide significance according to the recent established genome-wide significance threshold set for IBD mapping [31].

**Figure 1:**
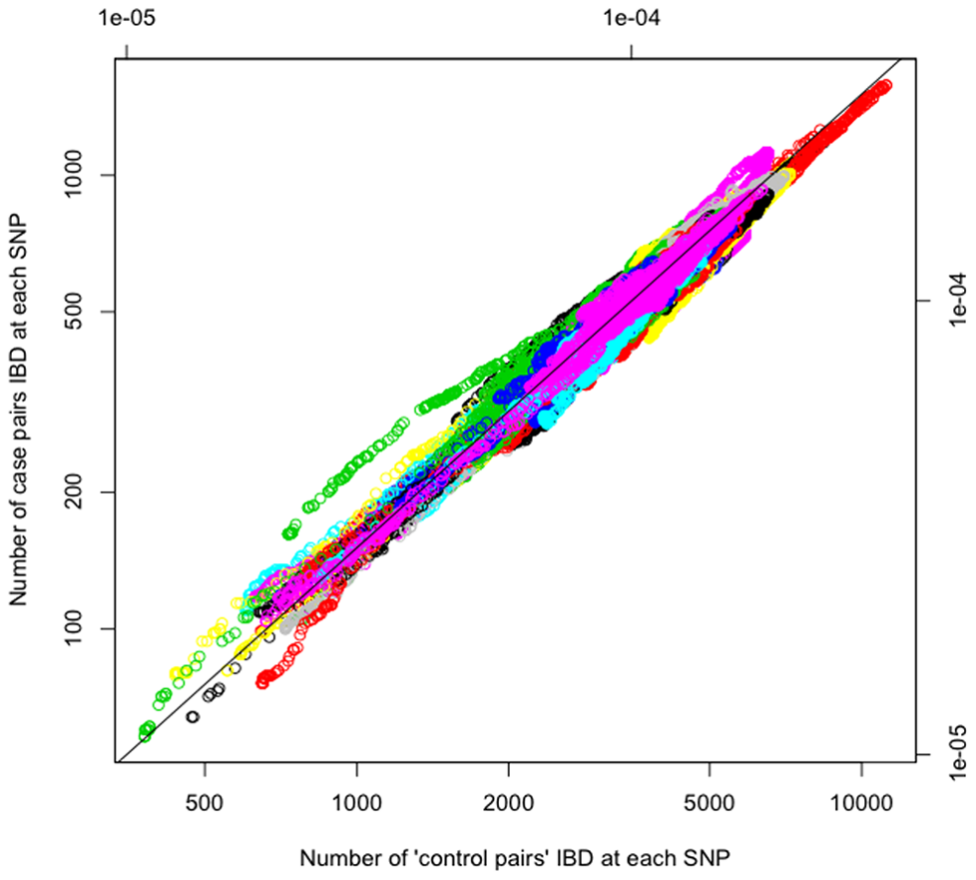
Plots of raw data of IBD with one point for each SNP. The green region is obvious outstanding from the black line, which indicates the proportion of case pairs in this region higher than that of control pairs. The black line represents where the proportion of case pairs equal to control pairs.

**Figure 1** is a scatterplot of case-pair sharing *yi* versus control-pair sharing *xi* as each of the 274,735 SNPs *i*. Using different colours to represent SNPs on different chromosomes, an outlier group of SNPs with relatively high case pair sharing on one chromosome stands out in green.

### Fitting, testing Poisson model and converting to LOD scores

**Figure 2:**
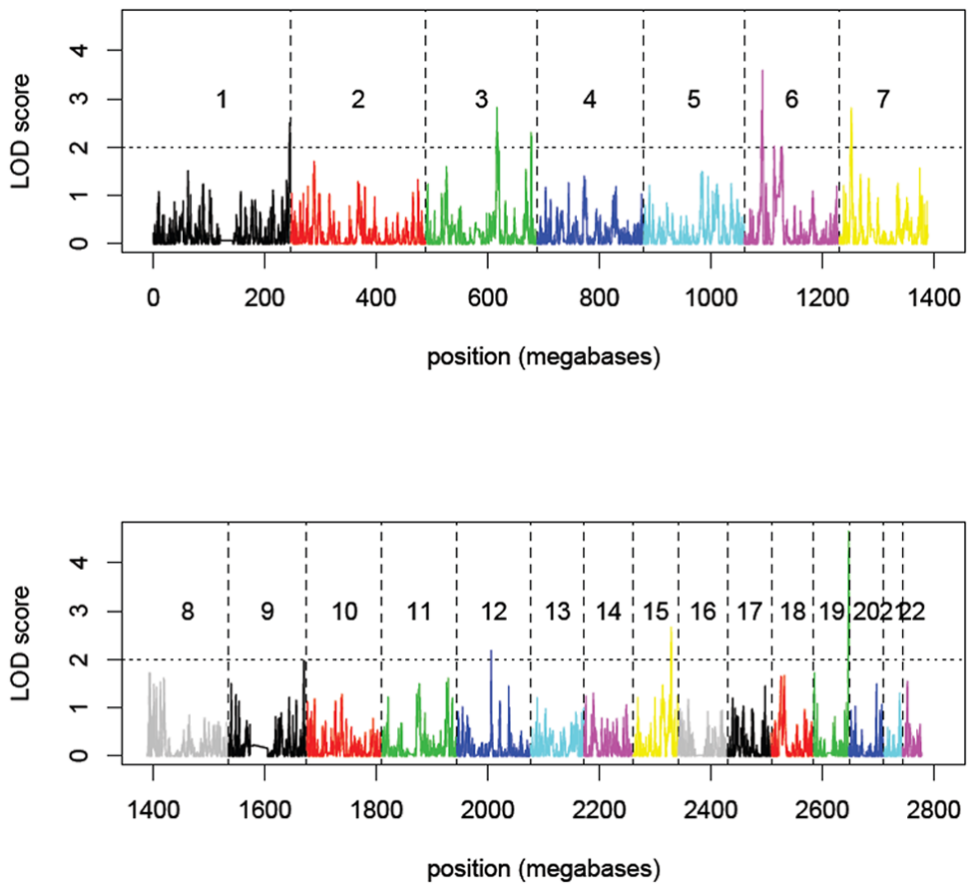
Plot of linkage scores along the whole genome with the IBD threshold of 3cM_1e-9 (shared haplotype segment >3 cM and haplotype probability p<10^−9^). Chromosome 19 has the strongest linkage signal (LOD = 4.65).

From examination of diagnostic plots (**Figure S2, S3, S4**), we found that the Poisson model provided the best fit for these data. **Figure 2** shows a plot of residuals from the Poisson model converted to LOD scores. The highest linkage signal, corresponding to the green outlier region in **Figure 1**, was observed on chromosome 19 with LOD = 4.65 and p = 1.9×10^−6^. As expected, a strong signal also was observed in the HLA region (LOD = 3.58; **Fig. 1**).

### Analysis of Linkage region on chromosome 19

The linkage region on chromosome 19 with LOD scores between 3.65 and 4.65 is around 900kb in length (Hg18 chr19: 62,529,738–63,437,743 bp) and corresponds to a cluster of zinc finger genes at 19q13.4, many of which have arisen by gene duplication. None of the genes in this region have been previously identified in published GWAS or associated with MS or autoimmune diseases.

The genes in this region were examined to identify candidate genes with putative roles, which could, impact on susceptibility to MS. Published microarray expression data [36], profiling gene expression in the human hippocampus over a broad developmental range, were downloaded from Gene Expression Omnibus [37], series number GSE25219. Gene summary data was analysed in Partek Genomics Suite version 6.6 (Partek Inc., St. Louis, MO, USA) to generate expression profiles across all developmental periods for genes in the linkage region. Many of these genes exhibit similar expression profiles with high expression in early time points and low expression after birth. To categorize this observed trend, samples from a number of early foetal time periods (3, 4 and 5) (described in [36]) were grouped together and compared with expression of grouped samples from periods 9, 10, 11, and 12. Differential expression of exon level probe sets between these two groups was then analysed. The data points corresponding to individual probe sets, and representing expression changes between these two developmental stages, were then aligned against the linkage region in a UCSC genome browser view. Differentially expressed probe sets were filtered using a false discovery rate adjusted p value cut off (1.53×10^−3^) equivalent to a p value threshold of 0.01 and a fold change minimum 1.5. Those probe sets that passed this threshold were plotted on the UCSC browser screen view [38]. Genomic locations for Affymetrix exon level probe sets within the linkage region were downloaded from the UCSC table browser [39] and used to construct a bedGraph file of expression changes. The green bars indicate a higher expression in foetal time points compared to later time points. Some fold changes for genes in this region are very high (4–6 fold higher in foetal than post birth).

**Figure 3:**
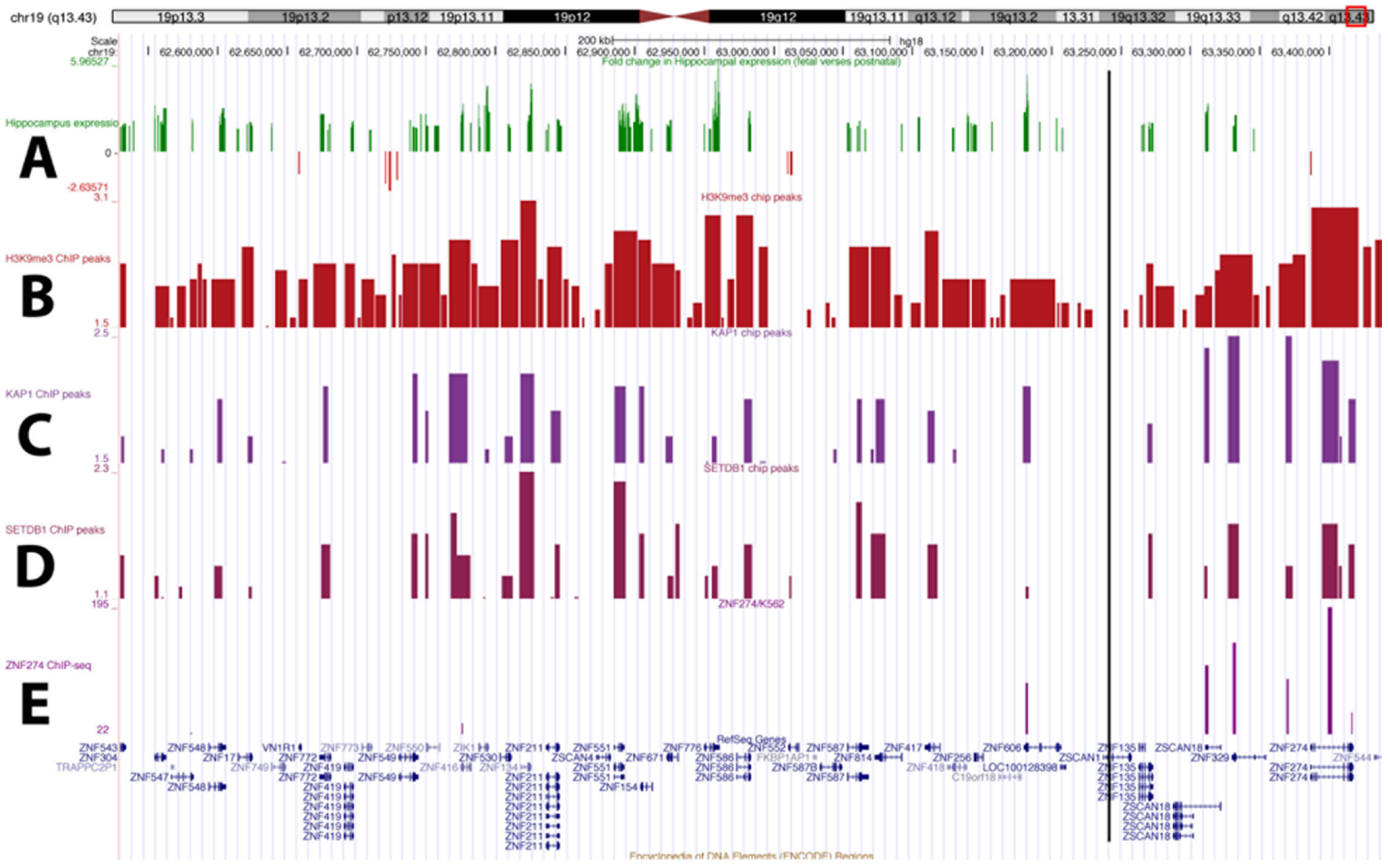
Screen shot from the UCSC genome browser illustrating expression regulation within the identified linkage region on Chromosome 19 (hg18) ( http://genome.ucsc.edu **).** Human Refseq gene models are shown at the bottom of the figure. Custom bedGraph tracks illustrating expression regulation, as described in the manuscript, are shown. From top to bottom: (A) exon level expression fold change in hippocampus (FDR adjusted p value <0.01 and fold change >1.5) between early fetal (periods 3,4 and 5) and postnatal (periods 9,10,11 and 12) from Kang et al 2011, green bars indicate increased expression in fetal compared with postnatal and red bars indicate decreased expression in fetal compared with postnatal. ChIP-chip binding patterns of (B) H3K9me3 (C) TRIM28/KAP1 (D) SETDB1 and (E) ChIP-seq binding pattern of ZNF274 in K562 cells. For the ChIP-chip data log2 (ratio) values reflecting the ChIP enrichments are plotted on the Y axis. For the ChIP-seq data the number of tags reflecting the ChIP enrichments are plotted on the Y axis. ChIP-chip and ChIP-seq data are from Frietze et al 2010 supplementary data. Chromosomal coordinates and relative position on the chromosome is illustrated in the idogram at the top of the figure. The position of SNP rs159870 is shown by a vertical black line.

Although little is known about the majority of genes in this region, *ZNF274* is a DNA binding protein involved in regulation of H3K9me3 methylation at the 3′ end of some ZNF genes by recruitment of the histone methyltransferase *SETDB1*, and the corepressor *TRIM28* (*KAP1*) [40]. To examine the pattern of H3K9me3 methylation in this region, genomic data on H3K9me3 methylation and *KAP1*, *SETDB1* and *ZNF274* binding in K562 cells [40] was used to make custom bedGraph files for visualization alongside the expression change with development (**Fig. 3**). From this data we observed that the vast majority of genes in this region with high foetal expression levels are marked by both H3K9me3 methylation and bound by *KAP1* and *SETDB1* at the 3′ end of the gene. A small number of genes are also bound at the 3′ end of the transcript by *ZNF274*. We also observed a pattern in the level of H3K9me3 methylation, with two maximum levels at about position 62,850,000 and 63,400,000 and trailing off at position 63,250,00. This bimodal pattern also occurs in the *KAP1* and *SETDB1* binding data and is even more apparent when viewing a wider view of the region. This position, marked in the figure by vertical black line, also corresponds with the position of rs159870 (chr19: 63239261) and there is break in synteny with rodent genomes in this zone.

### Comparison of IBD sharing among different populations

We next examined patterns of IBD sharing at the SNP with the highest LOD score on chromosome 19 (rs159872). We compared the proportion of IBD case pairs in different populations to determine whether there are particular populations that contribute to more IBD case pairs at this locus; and found the Tasmanian population has the highest proportion of IBD case pairs. When compared with all other combined Australian populations, the Tasmanian population significantly contributed more IBD case pairs at this locus (p = 0.004); and was significant when compared with all other combined non-Tasmanian populations (p = 5.44×10^−5^; **Table 2**).

**Table 2:** Comparison of IBD case pairs among different populations (rs159872 with the highest LOD score on chr19; LOD = 4.65).

Population	No. case	No. IBD case pairs	% IBD case pairs	p-value
TAS	308	7	14.80×10^−5^	Ref.
Mel	841	32	9.06×10^−5^	0.22
Newc	111	0	0.00	1.00
Syd	541	14	9.58×10^−5^	0.32
Other	32	0	0.00	1.00
AUS (non-TAS)	1525	46	3.96×10^−5^	0.004
NZ	540	14	9.62×10^−5^	0.32
US	879	22	5.70×10^−5^	0.033
Non-TAS	2944	82	1.89×10^−5^	5.44×10^−5^

* % IBD case pairs = IBD pairs/case×(case-1)/2; (Fisher's Exact Test).

### Networks of cases and controls sharing haplotypes on chromosome 19

**Figure 4:**
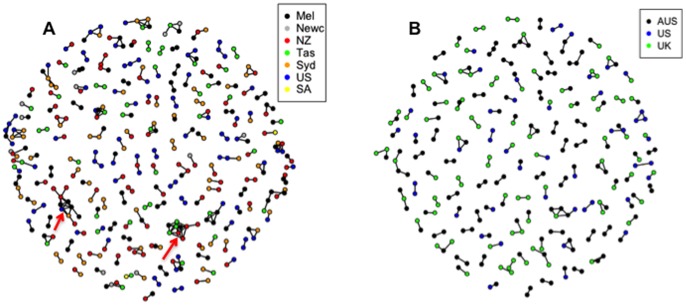
Networks of cases and controls sharing haplotypes IBD at the SNP with the highest LOD score on chromosome 19. (**A**) Networks of cases sharing haplotypes in common at the linkage region on chromosome 19. (**B**) Networks of controls sharing haplotypes in common at the linkage region on chromosome 19. Each dot represents an individual and each line connects pairs of individuals who share a haplotype. There are more big networks of cases sharing haplotypes than controls.

**Figure 4** shows networks of cases and controls sharing haplotypes IBD at the SNP with the highest LOD score on chromosome 19. The biggest cluster comprises 10 cases sharing a haplotype in which 4 cases were from Melbourne, 4 from New Zealand, 1 from Sydney and 1 from USA. Another big cluster includes 2 cases from Melbourne, 3 cases from Tasmania and 4 cases from New Zealand (**Fig. 4A**). More generally, there are more networks of cases sharing haplotypes than controls.

## Discussion

We have applied BEAGLE fastIBD for the detection of rare MS variants utilising a large-scale GWAS dataset. We identified a high linkage signal on chromosome 19 with a p-value of 1.9×10^−6^ (LOD = 4.65). In classical linkage analysis in small families, individuals are closely related and the segments of IBD tend to be fairly long (>10 cM) which are easier to detect and less independent than IBD mapping, the generally-accepted threshold for genome-wide significance is p = 2.0×10^−5^ [41]; while GWAS has more independent tests than IBD mapping, the threshold of genome-wide significance is around p = 5.0×10^−8^ [42], so the threshold of p-value for IBD mapping genome-wide significant should be between 5.0×10^−8^ and 2.0×10^−5^. Recently, researchers demonstrated that the genome-wide significance thresholds for IBD mapping depend on the IBD segment size detected or IBD generations [31]. For example, an IBD segment size of 2 cM corresponds to 25 generations and the genome-wide significance threshold is 2.0×10^−6^, while the segment size of 3.2 cM corresponds to 15 generations and the genome-wide significance threshold is 4.0×10^−6^ [31]. The strongest non-HLA linkage signal we detected in this study used a 3 cM segment size; which corresponds to 17 generations, thus the genome-wide significance threshold is between 4.0×10^−6^ and 2.0×10^−6^. As such, the linkage signal on chromosome 19, with a p-value of 1.9×10^−6^, was determined to be genome-wide significant.

### Causal relationship between genes in linkage region and MS

Most genes in this linkage region are zinc finger (ZNF) proteins of which 32 genes have been suggested to be transcriptional regulators [43] (http://genome.ucsc.edu/). Seven genes (*ZNF134, ZNF135, ZNF154, ZNF549, ZNF606, ZNF671* and *ZSCAN1*) in this region belong to the Krüppel family of ZNF genes. Only a few ZNF genes in this region have known vertebrate homologues and it includes a number of primate specific KRAB-ZNF genes [44]. In humans KRAB-ZNF genes number about 400 and make up the largest group of C2H2 transcription factors [45] which are typically expressed at low levels and involved in cell specific silencing and driving different cell lineages.

Detailed analysis of genes in this region did not reveal any direct links with MS. However examination of their expression profiles in published data revealed a shared early developmental CNS specific expression profile with 22 genes in this region being members of the expression module M20 described in [36], characterised by higher expression in all brain regions in early foetal time points followed by decreased expression prior to birth and very low expression thereafter. The M20 network of genes has a strong correlation with both neuronal differentiation and neuronal migration and a strong negative correlation with myelination [36].

Epigenetic mechanisms such as histone modification and DNA methylation are responsible for silencing many specific transcription factors including zinc finger genes, and the 3′ end of many ZNF genes are specifically covered by H3K9me3 [46]. The zinc finger gene *ZNF274*, located within the linkage region, is involved in gene silencing through recruitment of the histone methytransferase complex TRIM28 (KAP1)/SETDB1 to the 3′ end of specific ZNF genes [40]. Examination of H3K9me3, KAP1 and SETDB1 binding data, confirms that many of the genes in the linkage region are covered by H3K9me3 at their 3′end (**Fig. 3**). *ZNF274* also interacts with *p75NTR* and is predicted to play a role in programmed cell death during development [47]. A number of the genes in this area are also highly expressed in differentiated human neural cells compared to earlier stems cells (*ZNF549, ZNF324, ZNF548, ZNF264, ZNF671, ZSCAN1* and *ZSCAN18* are members of cluster A [48]). There is very little available evidence for involvement in immune cell activity for genes in this region. *ZNF304* is implicated in lymphocyte activation [49] and *ZNF274* has very high expression in activated eosinophils compared with other immune cell types [50]. Other genes in this region have relatively low expression and are not differentially regulated between immune cell types [50], as viewed in the immunological genome [51].

Together these findings suggest that many of the genes in this cluster may be involved in early differentiation of neuronal cells and potentially the silencing of genes required for myelination. Expression of ZNF genes is commonly detected in foetal brain and they are predicted to be involved in development of the nervous system, a KRAB zinc finger cluster on chromosome 8 has also been proposed to be involved in regulation of CNS development [52]. Although other clustered genes families have been shown to be co-expressed in cell types or tissues, previous studies have failed to identify coordinated expression of KRAB-ZNF gene clusters [44]. However earlier experiments did not examined the very early timepoints in CNS tissues included in the Kang dataset [36]. These expression profiles described in the M20 module are supported by two independent data set of both exon array level and RNA-seq expression data in early human CNS development available at the Allan Brain Atlas (http://developinghumanbrain.org/).

Thus this may be an example of a gene cluster of KRAB -ZNF genes exhibiting coordinated expression regulation, indicating the presence of a genomic regulatory block (GRB). Such regions are usually transcription factors controlled by highly conserved noncoding regions. Although the identification of GRBs remains difficult the evidence that we have collated is suggestive of two genomic regulatory blocks within the linkage region, interrupted at the position of SNP rs159870 where there is an absence of H3K9me3 methylation and a break in synteny (reviewed in [53]).

The underlying cause for susceptibility in this region could therefore be due, not to differences in a specific gene expression or protein product, but to differences in the tight expression regulation of a GRB. As mentioned above, many of the C2H2 zinc finger genes in this region have an expression profile consistent with silencing of genes required for myelination. Further analysis needs to be undertaken to examine if these genes are co-regulated in demyelination and remyelination as well as CNS developmental states. Unfortunately, due to the species specificity of many of the KRAB-ZNF genes and the absence of rodent homologues of genes in this region, data from non-human models of demyelination and remyelination may not be useful.

Ideally, re-sequencing is the next step to refine this potential signal further. Unfortunately, resequencing of the region would be complicated since there are many gene duplications in this linkage region.

For the SNP (rs159872) with the highest LOD score on chromosome 19, we hypothesise that there are some difference between cases and controls sharing haplotypes in the linkage region among different populations. We found the Tasmanian MS population has the highest proportion of case IBD sharing, significantly higher than non-Tasmanian combined populations as well as other non-Tasmanian combined Australian populations. While Tasmania has the highest prevalence of MS in Australia, it is generally agreed that this is primarily driven by environmental effects related to, sunlight and/or vitamin D [54]. However there is also a modest founder effect in Tasmania [55], which might result in an increase in MS susceptibility driven by rare variants IBD. Interestingly, we found there are more big networks of cases sharing haplotypes than controls, and one big case network comprises 3 Tasmanian cases, 4 New Zealand cases and 2 cases from Melbourne, which may indicate the potential causal variants or gene mutations exist in those big case networks. However, this SNP falls in a region of low/none methylation and correlates with a break in syteny, the significance of which is unclear.

### Technical considerations

Even though Beagle fastIBD is several orders of magnitude faster than Beagle IBD, IBD analysis remains moderately computationally intensive on a dataset of this size (8,977 individuals and 274,735 SNPs). For instance, on chromosome 2 with 22,607 SNPs, the computation time for each run was approximately 4.6 hours with memory requirement of 3.3 GB on 2 cores of a SGI Altix ICE 8200 HPC cluster computer node.

However, we also found IBD analysis limitations: it is only suited to discover rare variants if all variants act in the same direction in one gene. For example, the identified rare variants in *BRCA1* and *BRCA2* gene all increase risk of breast cancer [56], and the four rare variants identified in *IFIH1* gene all protect against type I diabetes [18]. If some rare variants increase risk while others in the same gene decrease risk then the signal in the region will be attenuated. In addition, we found IBD analysis is very sensitive to genotyping error, resulting in reducing signal strength. The linkage signal detected depends on a lot of markers or long haplotypes, containing up to hundreds of SNPs, a single error occurring in reading a single marker significantly reduces the signal. In our data, samples came from different GWAS using different genotyping chips in different locations, which at least in part, may decrease the potential signal strength from our study. Furthermore, resequencing would be complicated by gene duplication and repeat regions, since the linkage region detected in this study had many gene duplicates, thus replication in other independent dataset is needed.

The optimal method to detect rare disease-causing variants is whole genome sequencing of thousands of samples. When this becomes affordable, there will remain a role for IBD analysis to prioritize regions for follow-up analysis and minimize the massive multiple testing burden. Just as linkage analysis is now used to identify regions for follow-up in whole genome sequencing and exome sequencing of Mendelian disease families, and linkage analysis can be used to weight regions for GWA analysis [26].

In summary, we have applied IBD analysis to a large complex disease GWA dataset and identified a linkage signal with genome-wide significance, although it. While our most significant result is of equivocal significance, and lies in a region that is hard to validate via sequencing, we believe IBD analysis has considerable potential, particularly to help interpret whole-genome sequencing data in complex trait studies.

## Supporting Information

Figure S1 **Principal components analysis for the dataset.** Most individuals in the dataset are of predominantly northern European ancestry (right hand side), but some have southern European ancestry (left hand side) (one dot for each individual).
(TIF)

Figure S2 **Fitting Poisson model for the IBD data.** All the four real lines in these four modules fit well with the default lines, suggesting Poisson model is appropriate for this data. The residuals of the green region are higher than others.
(TIF)

Figure S3 **Fitting negative binomial model for the IBD data.** All the four real lines in these four modules fit not well with the default lines, suggesting negative binomial model is not suitable for this IBD data.
(TIF)

Figure S4 **Fitting linear model for the IBD data.** All the four real lines in these four modules fit not well with the default lines, suggesting linear model is not suitable for this IBD data.
(TIF)

SR_commands S1 1) Fitting and testing model for IBD data.
2) Plot of residuals from the Poisson model converted to LOD scores.
3) Network analysis.
(PDF)
