# Supplementary figures and images for: Identity-by-Descent Mapping to Detect Rare Variants Conferring Susceptibility to Multiple Sclerosis
Source: PLoS One. 2013 Mar 5;8(3):e56379. doi: 10.1371/journal.pone.0056379 (PMC3589405; doi:10.1371/journal.pone.0056379)

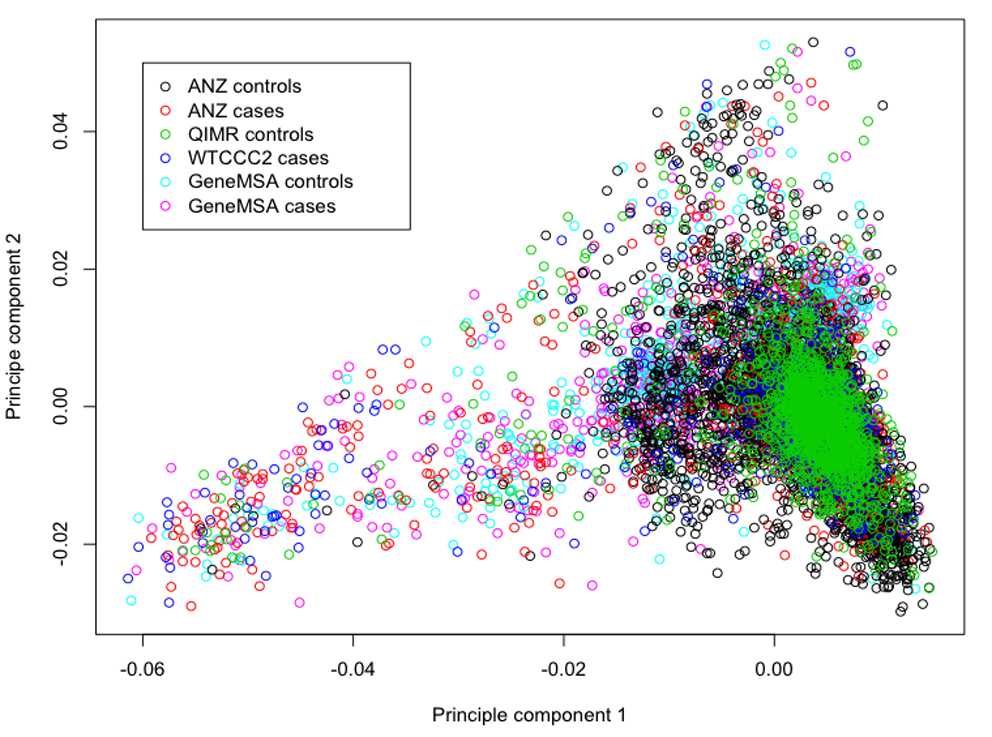

Supplement: Figure S1 — Principal components analysis for the dataset. Most individuals in the dataset are of predominantly northern European ancestry (right hand side), but some have southern European ancestry (left hand side) (one dot for each individual). (TIF) [file pone.0056379.s001.tif]

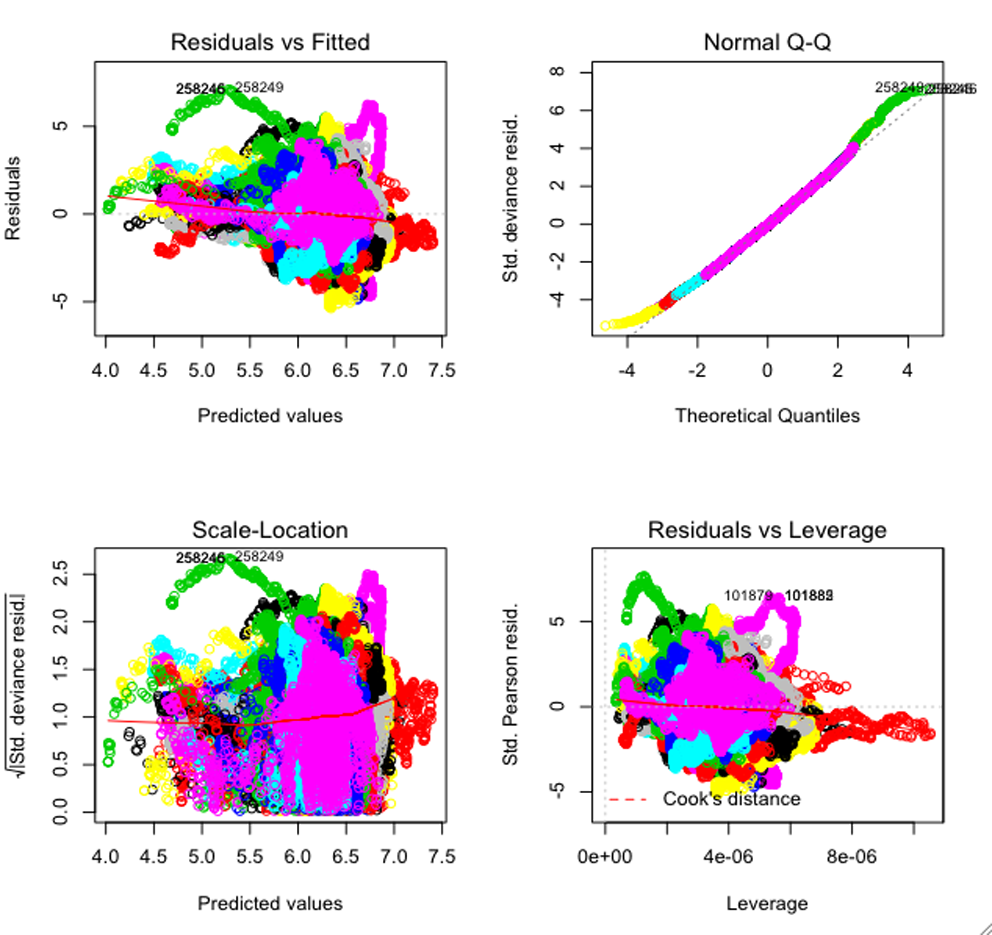

Supplement: Figure S2 — Fitting Poisson model for the IBD data. All the four real lines in these four modules fit well with the default lines, suggesting Poisson model is appropriate for this data. The residuals of the green region are higher than others. (TIF) [file pone.0056379.s002.tif]

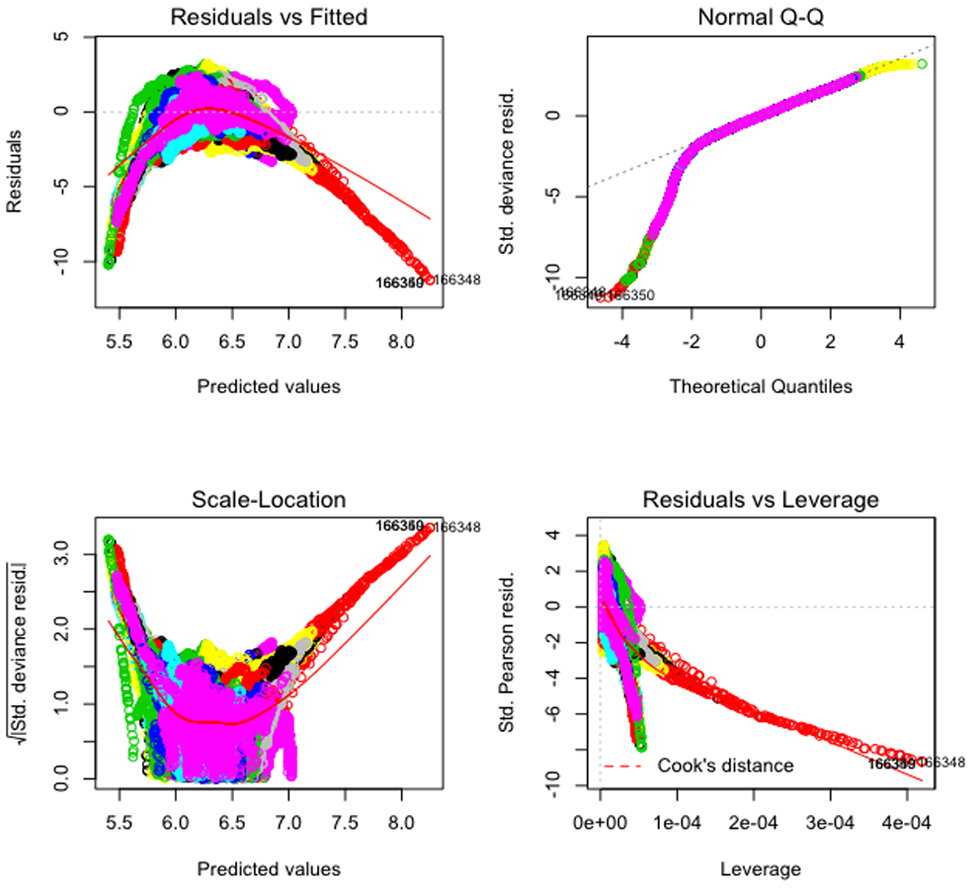

Supplement: Figure S3 — Fitting negative binomial model for the IBD data. All the four real lines in these four modules fit not well with the default lines, suggesting negative binomial model is not suitable for this IBD data. (TIF) [file pone.0056379.s003.tif]

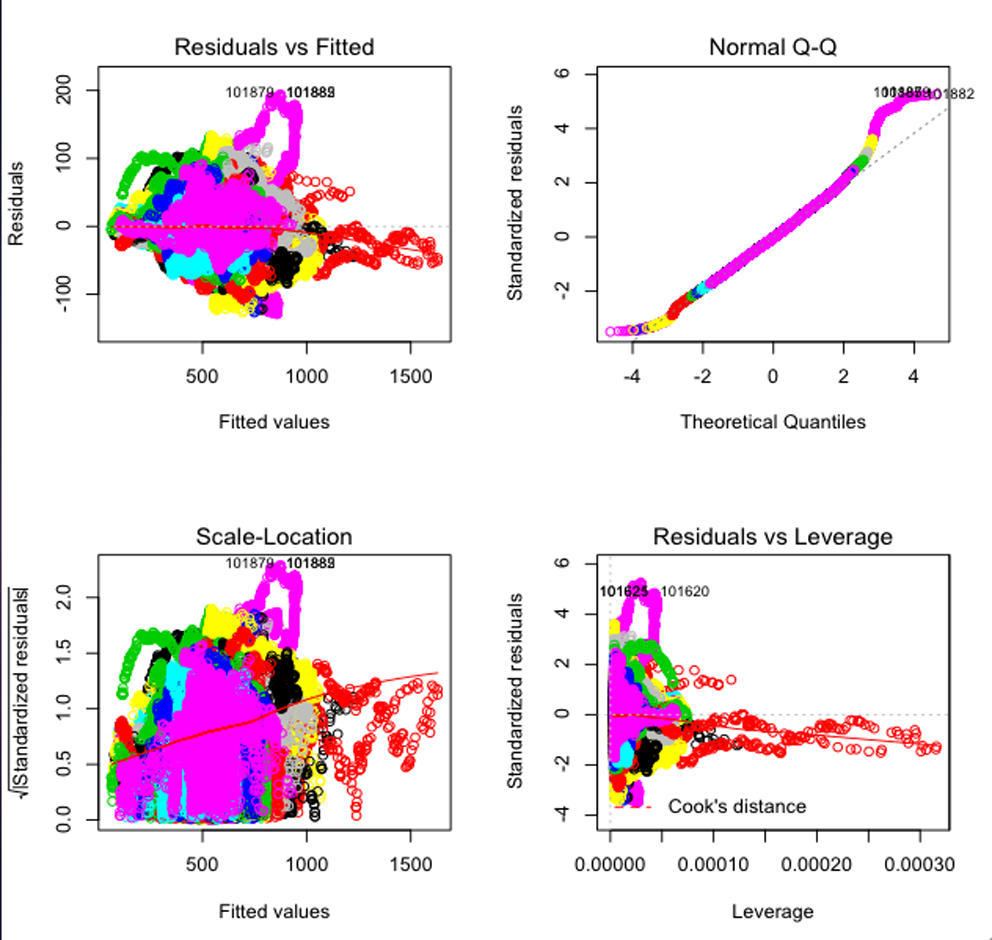

Supplement: Figure S4 — Fitting linear model for the IBD data. All the four real lines in these four modules fit not well with the default lines, suggesting linear model is not suitable for this IBD data. (TIF) [file pone.0056379.s004.tif]
