# Supplementary material for: Identity-by-Descent Mapping to Detect Rare Variants Conferring Susceptibility to Multiple Sclerosis
Source: PLoS One. 2013 Mar 5;8(3):e56379. doi: 10.1371/journal.pone.0056379 (PMC3589405; doi:10.1371/journal.pone.0056379)
Supplement: SR_commands S1 — 1) Fitting and testing model for IBD data. 2) Plot of residuals from the Poisson model converted to LOD scores. 3) Network analysis. (PDF) [file pone.0056379.s005.pdf]

SR_commands

1) Fitting and testing model for IBD data

```
library(MASS)
m1 <- glm.nb(case ~ control)
#nb = negative binomial regression
par(mfrow=c(2,2))
plot(m1,col=d$chr)

m2 <- lm(case ~ control)
#lm = linear regression
par(mfrow=c(2,2))
plot(m2,col=d$chr)

m3 <- glm(case ~ offset(log(control)),family=poisson)
#glm = poisson regression
par(mfrow=c(2,2))
plot(m3,col=d$chr)
```

2) Plot of residuals from the Poisson model converted to LOD scores

```
par(mfrow=c(2,1))
c <- 1
shift <- -(min(d$bp[d$chr==c]))/1000000
plot(d$bp[d$chr==c]/1000000+shift,LOD[d$chr==c],type="l",ylim=c(0,max(LOD)),xlim=c(0,1400),xla
 b="position (megabases)",ylab="LOD score",col=d$chr[d$chr==c])
lines(c(-100,1500),c(2,2),lty=3)
mid <- (min(d$bp[d$chr==c])+max(d$bp[d$chr==c]))/2000000+shift
text(mid,3,c)
for (c in 2:7) {
  shift <- shift+(max(d$bp[d$chr==c-1])-min(d$bp[d$chr==c]))/1000000
  lines(rep(shift+min(d$bp[d$chr==c])/1000000,2),c(-10,60),lty=2)
  lines(d$bp[d$chr==c]/1000000+shift,LOD[d$chr==c],col=d$chr[d$chr==c])
  mid <- (min(d$bp[d$chr==c])+max(d$b[d$chr==c]))/2000000+shift
  text(mid,3,c)
}
c <- 8
shift <- shift+(max(d$bp[d$chr==c-1])-min(d$bp[d$chr==c]))/1000000
plot(d$bp[d$chr==c]/1000000+shift,LOD[d$chr==c],type="l",ylim=c(0,max(LOD)),xlim=c(1400,2800)
 ,xlab="position (megabases)",ylab="LOD score",col=d$chr[d$chr==c])
lines(c(1300,3000),c(2,2),lty=3)
mid <- (min(d$bp[d$chr==c])+max(d$bp[d$chr==c]))/2000000+shift
text(mid,3,c)
for (c in 9:22) {
  shift <- shift+(max(d$bp[d$chr==c-1])-min(d$bp[d$chr==c]))/1000000
  lines(rep(shift+min(d$bp[d$chr==c])/1000000,2),c(-10,60),lty=2)
  lines(d$bp[d$chr==c]/1000000+shift,LOD[d$chr==c],col=d$chr[d$chr==c])
  mid <- (min(d$bp[d$chr==c])+max(d$bp[d$chr==c]))/2000000+shift
  text(mid,3,c)
}
```

3) Network analysis

```
library(network)
n <- read.table("3cM_1e-9_chr19.network",header=FALSE)
groups <- read.table("3cM_1e-9_chr19.grouplist",header=FALSE)
m <- as.matrix(n)
g <- network(m,directed=FALSE)
a <- plot.network(g,vertex.col=groups[,2],vertex.cex=0.7)
xmax <- max(a[,1])
ymax <- max(a[,2])
legend(xmax-10,ymax+10,legend=c("Mel","Newc","NZ","Tas","Syd","US","SA"),pch=rep(16,7),col=1:7)
```
